# Blood Vessel Injuries of the Fingers: A Clinical Comparison of One- and Two-Arterial Blood Supply

**DOI:** 10.3390/jcm12185889

**Published:** 2023-09-11

**Authors:** Nadjib Dastagir, Doha Obed, Florian Bucher, Shiar Murad, Khaled Dastagir, Peter M. Vogt

**Affiliations:** Department of Plastic, Aesthetic, Hand and Reconstructive Surgery, Hannover Medical School, 30625 Hannover, Germany; obed.doha@mh-hannover.de (D.O.); bucher.florian@mh-hannover.de (F.B.); murad.shiar@mh-hannover.de (S.M.); dastagir.khaled@mh-hannover.de (K.D.); vogt.peter@mh-hannover.de (P.M.V.)

**Keywords:** microsurgery, reconstructive surgery, plastic surgery

## Abstract

Traumatic finger injuries are very common in emergency medicine. When patients present with finger injuries, there is often damage to the vascular nerve bundles, which requires subsequent reconstruction. It is unknown if repairing a unilaterally injured artery affects patients’ recovery in a well-perfused finger. This retrospective cohort study compares the clinical outcomes of 11 patients with one-vessel supply (mean age 48.3 years; 7 males, 4 females) to 14 patients with two-vessel supply (mean age 44.5 years; 8 males, 6 females). The patient outcomes were assessed using patient questionnaires (Disabilities of Arm, Shoulder, and Hand (DASH), European Quality of Life 5 Dimensions 5 Level Version (EQ-5D-5L), and EuroQol visual analog scale (EQ-VAS)) and a clinical examination of hand function and imaging of circulatory efficiency. No significant changes were observed in the DASH, EQ-5D-5L, and EQ-VAS questionnaires. Clinical evaluation of hand function, measured by cold sensitivity, two-point discrimination, pain numerical analog scale, and grip strength also revealed no significant differences between cohorts. Blood flow measurements using thermal imaging revealed no effects on circulation in the affected digit. Collectively, the study finds reconstruction is not absolutely necessary when there is one intact digital artery as it is sufficient for healing and functional outcomes. We recommend finger artery reconstruction when both digital arteries are injured or if an immediate tension-free suture is possible.

## 1. Introduction

Traumatic injuries to the hand and upper extremity are very common cases in emergency medicine and require advanced microsurgical methods to reconstruct the nerves and vasculature in order to minimize functional losses [1]. In 2016, 26% of US emergency department injuries were related to injuries of the hand and upper extremity. The most frequent demographic for US hand and wrist emergency department injuries is adult males between 18 and 39 years of age [2]. Hand injuries can have a variety of causes, including occupation-, domestic-, and vehicle-related accidents [3]. Based on the cause of trauma, these injuries can also vary greatly in severity from mild lacerations to complex open injuries, which require extensive surgery on the nerves and vasculature [1]. Furthermore, in cases of skin discontinuity, where the skin is completely disrupted through all the layers, exploratory surgery is required, which can add to the complexity of the surgery and increase operative time. This process can identify additional injuries to the tendons, arteries, and nerves that are not otherwise superficially visible and might be missed. Finger reconstructive surgery aims to restore the digit’s length, sensation, and appearance in order to recapitulate the preinjury function and esthetic to the highest degree possible. The hand’s essential roles in communication, work, and psychological wellbeing underline the important contribution of reconstructive surgery for the patients’ functional outcomes and quality of life [4].

Treatment of a hand injury requires advanced surgical techniques to re-establish the innervation and the blood supply to promote proper wound healing. Repairing the hand’s nerves is often prioritized over arterial repair if the finger is already well perfused [5]. The digital nerves require a blood supply, and we know from previous studies that nerves are essential for regeneration in vertebrates [6]. If the blood supply of the finger is compromised to a significant extent, then it might cause downstream complications for nerve healing and function and compromise the patient’s ability to heal. A study examining how arterial reconstruction affects nerve healing did not find blood flow to be a significant factor [7]. However, it is generally believed that increasing the blood flow at an injury site will improve wound healing [8]. Vascular comorbidities, such as diabetes, atherosclerosis, and bleeding disorders, are also associated with significantly higher risks of complications following hand surgery [9], suggesting that a decreased blood supply may negatively influence wound healing.

Each finger receives its blood supply from two proper digital arteries [10]. Reconstruction of the arteries is clearly indicated when both arteries in the phalanx are damaged, thus eliminating the blood supply and causing ischemia. The time until reperfusion in the finger then becomes important as muscle tissue becomes irreversibly damaged as early as 6 h following warm ischemia, with other tissue typically following after 12 h [11]. Therefore, it is clear that when both proper digital arteries are damaged, reconstruction is indicated. However, the best management remains to be elucidated in the case of single-artery damage. It is unknown if reconstructing a unilaterally injured digit artery in a well-perfused finger improves functional outcomes following surgical reconstruction. This retrospective cohort study aimed to compare patient outcomes following single-finger injuries according to single- or double-artery blood supply following reconstruction. Using a combination of patient-reported outcomes, clinical examination, and imaging techniques, we studied how patients’ functional outcomes depend on digital artery reconstruction.

## 2. Materials and Methods

**Figure 1:**
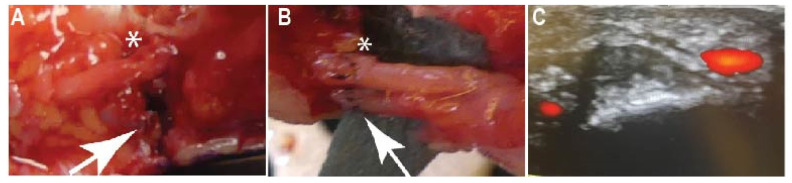
Representative images of the study cohorts. (**A**) A one-vessel blood supply patient without artery reconstruction (arrow) and with nerve reconstruction (asterisk). (**B**) A two-vessel blood supply patient with a reconstructed artery (arrow) and nerve (asterisk). (**C**) Doppler sonography of two intact arteries (red).

A total of 25 patients who had received treatment for a single finger injury from 2018 to 2021 were evaluated in this retrospective study. The patients were divided into two cohorts, those that received arterial reconstruction and had a two-vessel blood supply (n = 14) (2A) and those that did not have reconstruction due to the degree of damage to the artery (1A) (n = 11), (Figure 1). Patients were considered study-eligible if they had received finger reconstructive surgery of one digit at the flexor zone two. Eligibility criteria included being at least 18 years old, having a good understanding of the German language, and having a documented follow-up at least 12 months following the operation. The study was performed in accordance with the ethical standards of the Declaration of Helsinki and was approved by the local ethical review committee (No. 7887_BO_K_2018). All of the patients participating in the study did so voluntarily, and a consent form was signed and personally dated by the patients and surgeons. The surgeons made the decision for a one-vessel blood supply based on the extent of artery trauma and whether a tension-free artery reconstruction was feasible. In all cases, the nerves were coapted and the patency of the digital arteries was ensured by Doppler sonography.

Patient-reported outcomes were assessed using a variety of questionnaires. The Disabilities of the Arm, Shoulder, and Hand (DASH) questionnaire asks patients to score their physical function and symptoms following finger reconstruction on a scale of 0 to 100, with 0 representing no restriction and 100 representing maximum disability. General life quality was assessed using the European Quality of Life 5 Dimensions 5 Level Version (EQ-5D-5L), which uses five questions related to mobility, self-care, usual activities, pain/discomfort, and anxiety/depression, and the vertical EuroQol visual analog scale (EQ-VAS), which uses a numerical scale with 0 representing the lowest health profile and 100 representing the highest.

Clinical evaluation of hand and finger functionality was assessed using two-point discrimination (2PD), grip strength, and cold sensitivity. Two-point discrimination was measured using a caliper. Force measurements were made using a JAMAR dynamometer and using pinch strength test. Cold sensitivity was assessed by asking patients if they experienced sensitivity in their affected finger when exposed to cold air or cold water. Circulatory perfusion in the affected hand was measured using a forward-looking infrared (FLIR) thermal imaging camera and laser speckle contrast analysis (LASCA).

### Statistics

Statistical analysis between two groups was conducted using a *t*-test. All statistical analyses were performed using GraphPad Prism 9.2.0. Differences were considered statistically significant if *p* < 0.05.

## 3. Results

### 3.1. Patient Demographics

The mean age in the 2A cohort was 44.5 years (range 22–65) and 48.3 years (range 25–67) in the 1A cohort (Table 1). There were 8 males and 6 females in the 2A group and 7 males and 4 females in the 1A group. Both groups showed no postoperative complications. The mean follow-up time was 35.4 months (range 31–42) for the 2A patients and 41.5 months (range 32–47) for the 1A patients.

### 3.2. No Significant Differences in Patient-Reported Outcomes Based on Blood Supply

The patient-reported outcome questionnaires showed comparable functionality between the 2A and 1A groups (Table 2). The mean DASH was 43.89 ± 5.62 for the 2A group, which was similar to the results in the 1A group, which had a mean of 38.18 ± 4.25. The EQ-5D-5L and EQ-VAS scores also showed no significant differences between groups, with the 2A group having a mean of 0.88 ± 0.04 and 80.21 ± 4.08 and the 1A group having a mean of 0.92 ± 0.02 and 84 ± 3.90, indicating a similar patient-assessed health condition in both cohorts.

### 3.3. Clinical Examination Found No Functional Changes between One- and Two-Artery Blood Supply Patients

A clinical examination of the fingers also did not show significant changes between the 2A and 1A cohorts (Table 3). The mean two-point discrimination for the 2A group was 5.82 mm and 6.34 mm for the 1A group, demonstrating comparable nerve healing regardless of blood supply. The prevalence of cold sensitivity was also low in both groups with 43% reporting sensitivity in the 2A cohort and 45% in the 1A cohort. Pain, assessed by the numerical analog scale, showed no differences in stationary and working pain between the 2A and 1A cohorts. Functional testing of the grip strength compared to the healthy hand also demonstrated comparable results for the two groups with a mean JAMAR measurement of 69.51% for 2A and 66.34% for 1A. Pinch strength scores were also similar with a mean of 72.23% for the 2A group and 69.34% for the 1A group.

### 3.4. No Changes in the Affected Fingers’ Circulation and Perfusion

**Figure 2:**
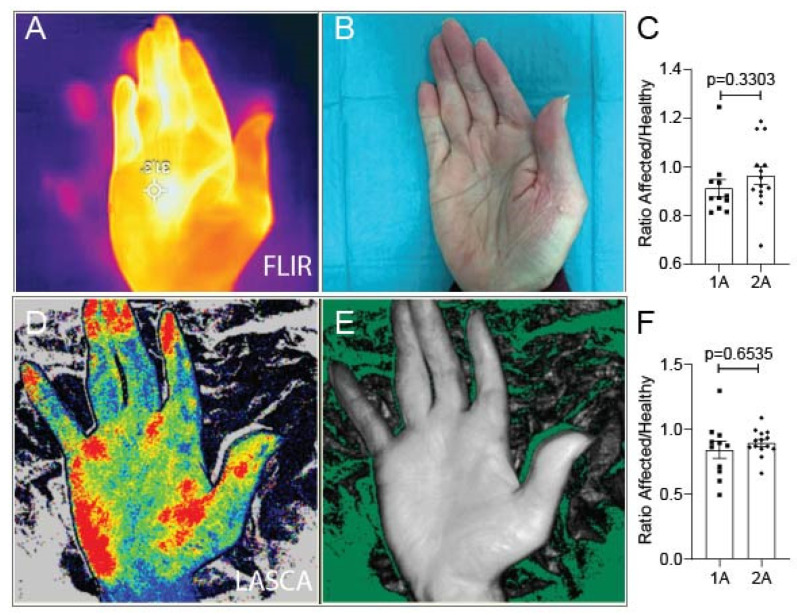
Representative images of circulatory perfusion. (**A**–**C**) FLIR imaging and quantification of the ratio of the affected hand’s circulation relative to the patient’s other healthy hand. (**D**–**F**) LASCA imaging and quantification of the ratio of the affected hand to healthy hand. Results are mean ± SEM. Square indicates one-artery patients, circle indicates two-artery patients.

Following a traumatic finger injury, the blood supply and sufficient perfusion must be verified. Physicians might be concerned that by only having one intact arterial supply, the circulation in the affected finger will be reduced. A circulatory assessment using FLIR and LASCA imaging revealed no significant differences between the 1A and 2A groups, demonstrating that one-artery blood supply was still sufficiently perfusing the digit (Figure 2).

## 4. Discussion

The hand is not only an important tool for work and recreation, but also for communication and self-expression [12]. Traumatic finger injuries can greatly influence hand, wrist, and upper limb function and affect patients’ daily lives [13,14]. It is therefore imperative that surgeons understand the factors involved in wound healing and reconstructive surgery in order to allow them to apprise patients of their decision making and to maximize patients’ functional outcomes following these injuries [4]. In our retrospective study, we examined the role of arterial blood supply in finger healing by comparing the results of a patient cohort with one-artery blood supply versus a cohort with two-artery blood supply. Depending on the extent of finger trauma, some patients arrive at the clinic with a unilateral injury where one artery is greatly damaged and cannot be reconstructed by tension-free suture, while the remaining artery is intact and capable of efficiently perfusing the finger. The decision for arterial reconstruction in such a case can result in extensive microsurgery leading to increased operating time and the potential for further complications. It is therefore important to understand how the digit’s blood supply contributes to wound healing and functional outcomes. Using a combination of patient questionnaires, clinical assessments, and circulatory imaging, we found that a one-vessel or two-vessel blood supply did not significantly impact healing and functional outcomes following unilateral finger injuries. Our finding sheds light on the role of arterial blood supply in finger healing and helps inform physicians when faced with patients that present with unilateral finger injuries.

The importance of vascularization in the healing process has been demonstrated in many studies [8,15,16,17]. Providing a sufficient blood supply during reconstructive surgery is known to be particularly important for the healing of soft tissues, bones, nerves, and tendons [18,19,20]. Throughout the wound healing process, vascularization plays a vital role by providing oxygen and nutrients to the damaged area and supporting the repair process [21]. The tissue oxygen partial pressure provided by blood perfusion is also important as it influences the bactericidal ability of neutrophils and collagen deposition. Thus, impaired blood supply could contribute to an increasing risk of wound infection and negatively affect the wound’s tensile strength [22,23]. It is therefore necessary to ensure efficient circulatory perfusion following traumatic finger injuries to minimize the risk of further complications, such as ischemia and necrosis.

The decision to reconstruct nerves is often prioritized before arteries, particularly if the finger is already well perfused, such as following a unilateral digital injury. Limiting the operating time to nerve reconstruction is particularly useful to prevent Wallerian degeneration, which can occur as quickly as 12 h following a nerve injury [7]. This study demonstrates that the decision to focus on nerve reconstruction rather than arterial reconstruction in the case of sufficient perfusion does not have major ramifications for functional outcomes. Importantly, in our patient cohorts, we did not find differences in nerve healing outcomes, which was measured using the two-point discrimination. Patients with one-artery blood supply had a mean score of 6.34 mm, while patients with a two-artery blood supply had a mean score of 5.82 mm, demonstrating comparable results. A similar study by Yildiran et al. (2020) focused on how arterial blood supply affects nerve healing. They found no significant differences in neuroma formation, two-point discrimination, or cold intolerance between the study groups that had an unrepaired artery and an intact artery group [7]. The results of our study concur with these findings. While blood supply is a useful factor in wound healing, we observed in our study that increased blood supply did not result in improved functional outcomes.

Based on our results, we hypothesize that a one- versus two-vessel blood supply does not influence healing due to collateral circulation compensating for the nonfunctioning vessel. Collateral recruitment has been well established in the past and is marked by the formation of collaterals following vessel occlusion to supplement the blood supply [24,25]. Following a traumatic injury, there is an inflammatory response that results in mild vasculitis, which in turn leads to collateral recruitment [26,27]. These collateral branches will then compensate for the damaged vessel and adapt to meet the vascular needs, which results in the development of the collateral circulation [28]. Our observation that a one-vessel blood supply did not result in impaired functional outcomes might be due to the development of collateral circulation forming from the intact, pre-existing artery. It would be interesting to characterize the role of collateral circulation in relation to finger healing after injury. Future work examining if changes in the development of the circulatory system following finger trauma influence functional outcomes would shed light on the importance of digital vasculature in healing.

Finger reconstruction surgery can be an onerous operation due to the complex characteristics of the digit’s anatomy and limited tissue availability. While advances in microsurgical approaches have greatly advanced the field, finger reconstruction remains a time-intensive operation relative to the amount of tissue treated. Artery reconstructions can be a particularly challenging microsurgery based on the cause of injury. For example, arterial reconstruction following avulsion injury can be difficult to impossible depending on the extent of the injury. Following an avulsion injury, there is often extensive damage to the nerves and vessels, and in contrast to a guillotine-type injury, they are located at varying distances relative to the level of skin avulsion (https://oali.com/wp-content/uploads/2020/11/100.pdf (accessed on 3 July 2023)). This can further limit the amount of viable tissue present for reconstruction compared to a guillotine injury and increases the likelihood of damage to the arterial blood supply. Crush-force injuries can also increase the complication of arterial reconstruction due to the trauma often affecting a larger tissue area beyond the obvious area of insult. As a result, extensive exploratory surgery may be needed to identify all trauma-related injuries and re-establish innervation and vascular perfusion. The time needed to perform such complex microsurgery also prolongs the operating time due to the necessity for extensive nerve reconstruction. Consequently, it is important to identify the factors that have a demonstrated correlation with patients’ functional outcomes to inform surgical decision making and operative priorities.

Patients that were included in this study had no subsequent complications following the arterial reconstruction. It is also important to note that patient comorbidities can greatly influence the extent of digital healing. For example, tobacco smoking has been shown to delay bone union and prolong healing time [29]. This can be a particular concern during the healing process of hand injuries as cigarette smoke can reduce the digital blood flow by as much as 42% [8,30]. When treating patients with traumatic finger injuries, it is important to consider their medical history and comorbidities that might affect their healing and recovery. It is also important to note that dysvascular finger injuries can manifest in various forms. In one interesting case report from Bouz et al. (2021), a patient was reported presenting with an ischemic finger following a closed ring avulsion injury [31]. Surgical exploration found isolated disruption in both digital arteries, while both digital nerves remained functional. Such a case is a useful reminder for hand surgeons that dysvascular digits may present with a diverse range of symptoms and extensive vascular injury remains possible without clear visible trauma.

As techniques in clinical medicine advance, surgeons are able to tailor their decision making to best meet their patients’ unique needs and ideally maximize patients’ postoperative satisfaction. This enhanced decision making is particularly important in hand surgery as these operations can often have drastic impacts on the patient. Due to the high percentage of hand injuries being caused by trauma, these unpredicted events can lead to major changes in life quality and can have psychological effects on the patient’s wellbeing. One study examining the effects of hand surgery on patients’ lives found 91.5% of study patients had to take time off from work and 58.6% relied on a significant other as their primary caregiver [32]. It is therefore imperative that when patients undergo finger reconstruction surgery, they understand the healing process and the surgeon is able to make the decision that will maximize their postoperative quality of life. In our patient group, we did not observe any significant changes in patient-reported outcome scores using the DASH, EQ-5D-5L, and EQ-VAS scores. The results of these questionnaires demonstrate comparable self-perceived health profiles in these cohorts. This knowledge is useful as it helps guide clinicians’ decision making during finger reconstruction based on the patients’ unique cases and helps clinicians determine the type of microsurgery that will offer the patients the highest functional outcomes.

There are some limitations to the results of this study. As a retrospective study, the slight variations in patient follow-up times can influence the reported results. Additionally, this study is based on the results of a single institution. It would be ideal to expand on this work in a more heterogenous population to account for differences in patient demographics and multi-institution differences. Such further studies would be helpful to identify additional trends related to blood supply and finger healing outcomes following traumatic injuries. Overall, this study helps raise awareness of the role of vascular blood supply in finger reconstruction and can be further examined in a larger patient population in the future.

While our results indicate that one- versus two-artery blood supply does not significantly affect the functional outcomes of traumatic finger injuries, we still recommend arterial reconstruction when possible. The patient continues to have the risk of recurring injury in the affected hand, and by repairing both arteries, it maximizes perfusion in the finger. The reconstruction of the artery will also help maintain anatomical structure and integrity for the patient. While we did not find significant differences between the one- and two-supply groups, reconstruction of both arteries gives the patient the highest likelihood of functional and self-reported postoperative recovery.

## 5. Conclusions

Our study reveals that there are no major differences in functional outcomes based on one- or two-vessel blood supply following traumatic finger injury. The DASH, EQ-5D-5L, and EQ-VAS questionnaires showed comparable scores between both examined cohorts. All other parameters measured, including clinical assessment of grip strength, pain numerical analog scale, cold sensitivity, and circulatory imaging did not show significant differences between the groups. Therefore, when a patient has a unilateral injury in a well-perfused finger, arterial reconstruction is not absolutely necessary for healing; however, we recommend reconstruction of both arteries when possible.

## Figures and Tables

**Table 1:** Patient Demographics.

Characteristic	2A	1A
Mean age (years)	44.5	48.3
Mean follow-up (months)	35.4	41.5
Complications	0	0
Male	8	7
Female	6	4

**Table 2:** Summary of Patient-Reported Outcome Questionnaires.

	Mean ± SEM		*p* Value
	2A	1A	
DASH Score	43.89 ± 5.62	38.18 ± 4.25	0.426
EQ-5D-5L Score	0.88 ± 0.04	0.92 ± 0.02	0.370
EQ-VAS Score	80.21 ± 4.08	84 ± 3.90	0.509

**Table 3:** Summary of Clinical Examination Results.

	Mean ± SEM		*p* Value
	2A	1A	
NAS at rest	1.14 ± 0.14	1.36 ± 0.20	0.385
NAS working	3.50 ± 0.61	2.73 ± 0.62	0.383
JAMAR (%)	69.51 ± 6.79	66.34 ± 7.48	0.756
Pinch strength (%)	72.23 ± 3.79	69.34 ± 9.79	0.433
2PD	5.82 ± 1.12	6.34 ± 1.27	0.559
Cold Sensitivity	43%	45%	

## Data Availability

The data presented in this study are available on request from the corresponding author. The data are not publicly available due to patient privacy.
